# Lower liver cancer risk with antiviral therapy in chronic hepatitis B patients with normal to minimally elevated ALT and no cirrhosis

**DOI:** 10.1097/MD.0000000000004433

**Published:** 2016-08-07

**Authors:** Joseph K. Hoang, Hwai-I Yang, An Le, Nghia H. Nguyen, Derek Lin, Vinh D. Vu, Kevin Chaung, Vincent Nguyen, Huy N. Trinh, Jiayi Li, Jian Q. Zhang, Chien-Jen Chen, Mindie H. Nguyen

**Affiliations:** a Division of Gastroenterology and Hepatology, Stanford University Medical Center, Stanford, California, USA; b Genomics Research Center, Academia Sinica; c Institute of Clinical Medicine, National Yang-Ming University, Taipei, Taiwan; d Stanford Cancer Institute, Stanford Medicine, Stanford; e Department of Internal Medicine, University of California, San Diego; f Department of Medicine, Stanford University Medical Center, Stanford; g San Jose Gastroenterology, San Jose; h Gastroenterology, Palo Alto Medical Foundation, Mountain View; i Primary Care, Chinese Hospital, San Francisco, California, USA; j Graduate Institute of Epidemiology and Preventive Medicine, National Taiwan University, Taipei, Taiwan.

**Keywords:** ALT, antivirals, HBV DNA, hepatocellular carcinoma, REACH-B

## Abstract

For chronic hepatitis B (CHB), alanine aminotransferase (ALT) ≥2 × upper limit of normal (ULN) is often used as a major criteria to initiate treatment in absence of cirrhosis, though patients with lower ALT may not be free from future risk of hepatocellular carcinoma (HCC). We aimed to examine the effect of antiviral therapy on HCC incidence based on ALT levels.

We performed a retrospective study on 3665 patients consisting of United States and Taiwanese REVEAL-HBV cohort who were consecutive, treatment-naïve, noncirrhotic CHB patients aged ≥40 years. Patients were categorized by ALT cutoffs (≥2 × ULN vs <2 × ULN) and subgrouped by treatment status. Kaplan–Meier and Cox proportional hazards models were used to calculate cumulative incidence and hazard ratio (HR) of HCC adjusting for REACH-B scores.

A total of 202 patients developed HCC. Antiviral treatment significantly reduced HCC risk: HR 0.24, 95% confidence interval 0.10–0.58; *P* = 0.001. HCC incidence per 100,000 person-years was significantly higher in untreated versus treated patients, even for those with ALT < 2 × ULN: 314.46 versus 0 per 100,000 person-years, *P* = 0.0042. For patients with Hepatitis B Virus (HBV) Deoxyribonucleic Acid (DNA) ≥ 2000 IU/mL, the number-needed-to-treat (NNT) were 15 and 14 to prevent 1 incident HCC at year 10 for patients with ALT < 2 × ULN and ≥2 × ULN, respectively.

After adjustment by REACH-B score, antiviral treatment significantly decreased HCC incidence even in patients with ALT < 2 × ULN. NNT to prevent 1 incident HCC after 10 years of therapy was low (14–15) in patients with mildly elevated HBV DNA ≥ 2000 IU/mL regardless of ALT levels.

## 1 Introduction

Chronic hepatitis B (CHB) is a significant worldwide health problem with an estimated 240 million people infected.^[1]^ A significant majority of affected individuals are from the Asia-Pacific region, and infection is often transmitted perinatally or during early childhood.^[2,3]^ Patients with CHB are at significant risk for developing cirrhosis, end-stage liver disease (ESLD), and hepatocellular carcinoma (HCC). CHB-related mortality is estimated to be half a million deaths each year.^[4]^

The progression of CHB is a multistage, multifactorial process involving interactions among host, environmental, and viral factors.^[5]^ Pivotal studies on CHB have identified many important virological and host predictors of HCC: hepatitis B e antigen (HBeAg) serostatus, alanine aminotransferase (ALT) levels, and HBV DNA levels.^[6–8]^ ALT is a sensitive indicator of hepatocellular injury and, in conjunction with HBV DNA levels, has routinely been used in clinical practice to identify patients who would benefit for anti-CHB treatment.^[9,10]^

While there are many potent and safe medications for the treatment of CHB, identifying those who are at risk for developing liver-related sequelae from CHB and should receive treatment is not clear.^[9–12]^ In the United States, there are 2 major treatment guidelines (US Panel Algorithm and American Association for the Study of Liver Diseases [AASLD]) with different opinions for when to initiate treatment.^[9,13,14]^ Recently updated AASLD guidelines 2015 recommend ALT ≥ 2 × ULN; ULN defined as ≥30 IU/L for men and ≥19 IU/L for women as a threshold for antiviral therapy in patients with elevated HBV DNA > 20,000 IU/mL, since data suggest that anti-CHB therapy is more effective above this level.^[13,14,15]^ In patients with ALT 1 to 2 × ULN, the AASLD guideline recommends an individualized approach. However, recent studies in patients with normal to minimally elevated ALT levels suggest that this may be too conservative of a recommendation, since patients may still be at risk for liver-related sequelae from CHB.^[16]^ There has been no study to date assessing the effect of antiviral therapy on HCC risk in patients with normal or minimally elevated ALT level but elevated HBV DNA levels.^[17,18]^

Our aim is to examine effect of antiviral therapy on the incidence of HCC in patients who did not yet have liver cirrhosis, were 40 years or older including those with low ALT levels. We adjusted background risks using the REACH-B predictive score, a previously validated composite 17-point HCC risk-prediction score.^[8,19,20]^

## 2 Methods

### 2.1 Study design

We conducted a retrospective cohort study of consecutive patients with CHB seen at several medical clinics in Northern California from 1991 to 2014 and patients from the previously described Taiwanese cohort, REVEAL-HBV, recruited from 1991 to 1992, who met the following inclusion criteria. Inclusion criteria were noncirrhotic patients aged 40 years or more. Exclusion criteria were coinfection with hepatitis C virus (HCV), hepatitis D, or HIV, HCC at presentation or within 6 months, cirrhosis at presentation or within 2 years. A total of 3665 patients who met study inclusion and exclusion criteria were included in the study analysis.

Treatment for CHB included any of the Food and Drug Administration (FDA)-approved agents: lamivudine, adefovir, entecavir, telbivudine, tenofovir, interferon, or combination therapy. Indications for antiviral therapy were generally based on either the AASLD or US Panel guidelines, in which ALT threshold for treatment can be ALT > 2 × ULN or ALT > ULN only, respectively.

### 2.2 Data collection

United States patient baseline characteristics such as age, gender, ALT, HBV DNA levels, treatment data, and HCC diagnosis were determined by individual patient chart review using a case report form developed for this study. Using AASLD criteria (2005 and 2011), HCC diagnosis was determined using biopsy reports or radiographic evidence from computed tomography (CT), magnetic resonance imaging (MRI), together with tumor markers, such as alpha-fetoprotein.^[21,22]^ Similarly, cirrhosis diagnosis was determined by reviewing histological, clinical, imaging data, or evidence of ascites, encephalopathy, splenomegaly, varices, or thrombocytopenia (platelet < 120,000/μL) accompanied by splenomegaly. In addition, selected patients did not have cirrhosis and did not have hepatic decompensation.^[23]^ The ascertainment of HCC cases in the REVEAL cohort was similar and also based on histopathological evidence, imaging techniques including CT, MRI, angiogram, and alpha-fetoprotein levels. Cirrhosis was ascertained using the abdominal ultrasonography with a quantitative scoring system based on features of liver surface, liver parenchymal texture, intrahepatic blood vessel size, and splenic size.

### 2.3 REVEAL-HBV cohort

The REVEAL-HBV is a community-based cohort of patients aged 30 to 65 years recruited from 1991 to 1992 from 7 townships in Taiwan who were hepatitis B surface antigen (HBsAg) seropositive and anti-HCV seronegative.^[20]^ Patients received no antiviral therapy as was local care standard at the time. Baseline characteristics at study entry including age, sex, HBeAg seropositivity, HBV DNA, ALT level, and cirrhosis status were collected.

### 2.4 REACH-B risk score for development of HCC

The REACH-B HCC predictive risk score is a composite 17-point assessment based on 5 clinical, laboratory, and virologic parameters (gender, age, HBeAg status, ALT levels, and HBV DNA levels) from the REVEAL-HBV cohort in Taiwan.^[8,19]^ The purpose of this score was to provide clinicians with a simple clinical tool to predict a patient's risk (3-year, 5-year, and 10-year) of developing HCC and help identify those who may need treatment and/or HCC surveillance.^[19]^ This risk score has been externally validated.^[19,24]^

### 2.5 Statistical analysis

For the descriptive analysis, we used proportions to describe categorical variables and mean with standard deviation, or median with interquartile range, to describe continuous variables where appropriate. Differences between groups were qualitatively and quantitatively analyzed using Chi-square test for categorical variables and Student *t* test for continuous variables. Nonparametric tests, Fisher exact test for categorical variables, and Wilcoxon rank sum test for continuous variables were applied when assumptions of normality were not met. Our primary endpoint was the development of HCC during follow-up. REACH-B scores were calculated for all patients. We used a Cox proportional hazard regression model to estimate hazard ratios (HRs) and accompanying 95% confidence intervals (CIs) for the development of HCC for antiviral treatment and with adjustment for REACH-B score as a continuous variable. On sensitivity analyses, we estimated HR (95% CI) and crude incidence of HCC with antiviral treatment in patients with HBV DNA > 2000 IU/mL and stratified by ALT (≥2 × ULN: treated vs untreated and <2 × ULN: treated vs untreated). We also calculated the number-needed-to-treat (NNT) to estimate the number of patients who needed to be treated to prevent 1 additional HCC occurrence and in accordance with the method of Altman et al^[25]^ in a survival setting with time-varying effects. All statistical tests were two-sided with a two-sided *P* < 0.05 as the definition of statistical significance. We performed our statistical analyses with STATA 11 (Stata Corporation, College Station, TX) and SAS software (SAS Institute, Cary, NC).

This study was approved by the Institutional Review Board at Stanford University (Stanford, CA) and Academia Sinica (Taipei, Taiwan).

## 3 Results

### 3.1 Patient characteristics

A total of 3665 patients met study inclusion criteria. Table 1 describes the baseline characteristics of the whole study cohort categorized by ALT cutoffs and by treatment status. Almost all patients were of Asian descent (98.2%). The REVEAL cohort did not receive antiviral therapy during the study period, while United States cohort contained treated and untreated patients. A total of 548 patients (15.0%) received antiviral therapy. The majority of patients received either entecavir or tenofovir monotherapy or in combination (82%) with adefovir, lamivudine, and pegylated interferon making up the remaining minority. Overall median follow-up time was 8.9 years. A total of 202 patients in the cohort developed HCC (7 treated vs 195 untreated).

**Table 1:**
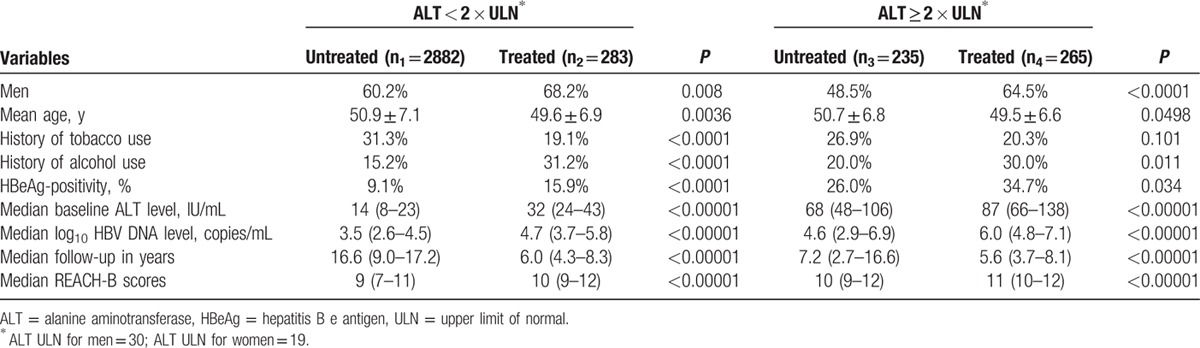
Baseline characteristics by alanine aminotransferase levels.

Patients in the treated versus untreated groups (regardless of ALT cutoffs) were more likely to be men, with lower tobacco usage, higher alcohol usage, higher rates of HBeAg-positivity, higher median baseline ALT levels, higher median HBV DNA levels, and higher median REACH-B scores (Table 1).

**Figure 1:**
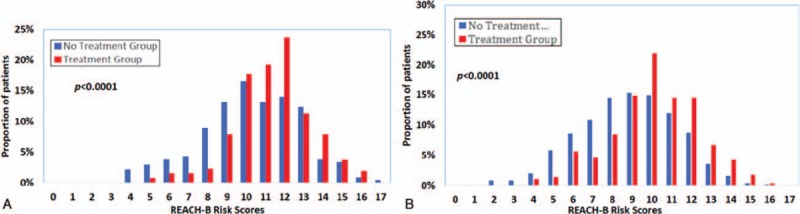
(A) Distributions of REACH-B risk scores for the treated cohort compared to untreated cohort in patients with aminiotransferase (ALT) ≥2 × upper limit of normal (ULN). (B) Distributions of REACH-B risk scores for the treated cohort compared to untreated cohort in patients with ALT < 2 × ULN.

Frequency distributions of REACH-B scores in the 4 groups of patients by ALT and treatment status are described in Fig. 1. REACH-B scores were higher in treated group than untreated group in patients with ALT levels of ≥2 × ULN (Fig. 1A) as well as in those with ALT < 2 × ULN (Fig. 1B).

### 3.2 Effect of antiviral therapy on HCC risk in overall cohort of noncirrhotic patients

As shown in Table 2, the incidence rate of HCC per 100,000 person-years was 208.90 for US-Treated group, 438.52 for US-Untreated group, and 488.39 for REVEAL-Untreated group.

**Table 2:**
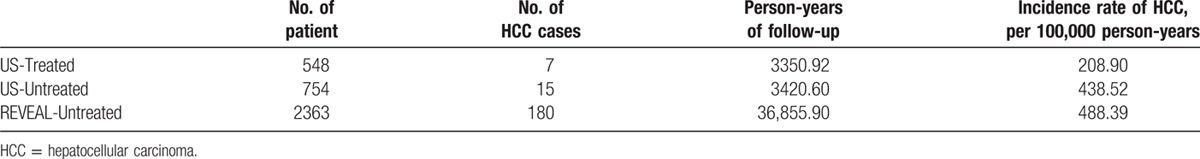
Incidence rates of hepatocellular carcinoma in patients by treatment status.

In Table 3, antiviral therapy was significantly and strongly associated with reduction of HCC risk when the US-Treated group was compared to the US-Untreated group alone (adjusted HR = 0.24 with 95% CI of 0.10–0.58, *P* = 0.0017), when the US-Treated group was compared to the REVEAL-Untreated group alone (adjusted HR = 0.32, 95% CI 0.15–0.70, *P* = 0.0042), as well as when the US-Treated group was compared to the combined US-REVEAL-Untreated group (adjusted HR = 0.31, 95% CI 0.14–0.67, *P* = 0.0027). In all 3 models, REACH-B score was a significant independent predictor of HCC, with each point increase in REACH-B score associated with a 36% increase in the development of HCC.

**Table 3:**
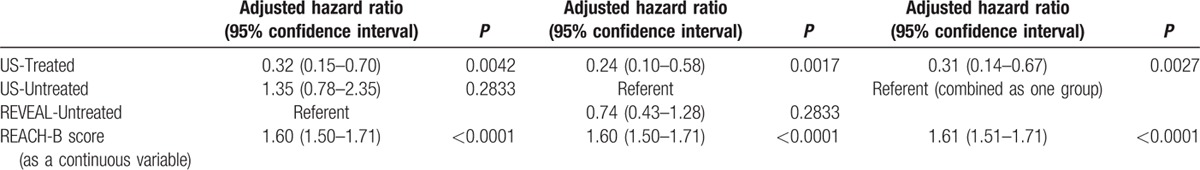
Incidence rates of hepatocellular carcinoma with adjustment for REACH-B score.

Table 4 shows incidence of HCC stratified by REACH-B scores. After adjusting for REACH-B, the HR for scores ≤10 was 1.34 with 95% CI 1.12 to1.60 (*P* = 0.001), scores 11 to 13 had HR 1.40 with 95% CI 0.92 to 2.12 (*P* = 0.114), and scores ≥13 had HR 1.55 with 95% CI 1.18 to 2.03 (*P* = 0.001).

**Table 4:**
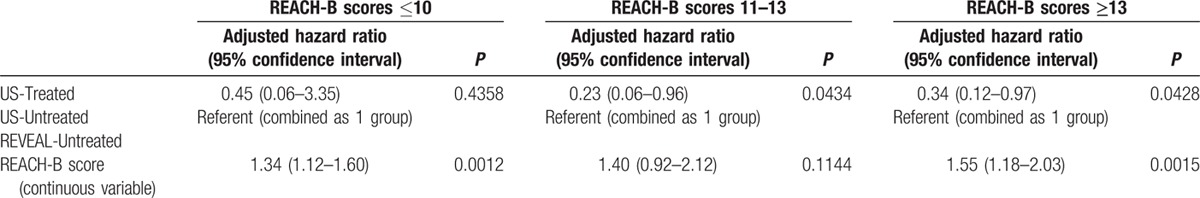
Incidence of hepatocellular carcinoma in patients with or without antiviral therapy stratified by REACH-B scores.

### 3.3 Cumulative incidence of HCC and effect of antiviral treatment, by ALT and HBV DNA levels

#### 3.3.1 ALT ≥ 2 × ULN

Crude HCC incidence was 484.15 cases per 100,000 person-years in untreated patients versus 208.90 cases per 100,000 person-years in treated patients. Patients who received treatment had a significantly lower cumulative incidence of HCC compared to untreated patients (*P* = 0.0291). Following adjustment for REACH-B scores, HCC risk was significantly reduced in patients who received treatment versus those who did not (HR 0.31, 95% CI 0.14–0.66, *P* = 0.003). The cumulative probability of developing HCC was 4.40% in untreated group versus 1.79% in treated group, yielding a NNT of 38 patients at year 5 overall (Table 5). At 10-year follow-up, the cumulative probability of developing HCC was 11.82% in the untreated group, while the cumulative probability of developing HCC was 6.12% in the treated group, which yielded a NNT of 18 patients to prevent 1 incident HCC case at year 10 overall (Table 6).

**Table 5:**
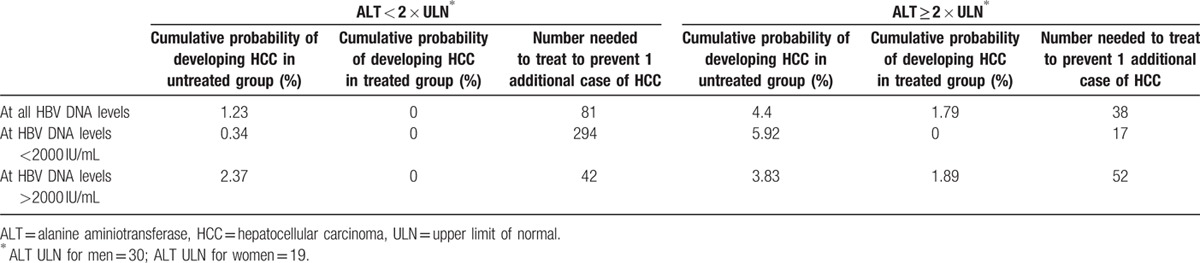
Number-needed-to-treat to prevent 1 incident case of hepatocellular carcinoma at 5 years of follow-up.

**Table 6:**
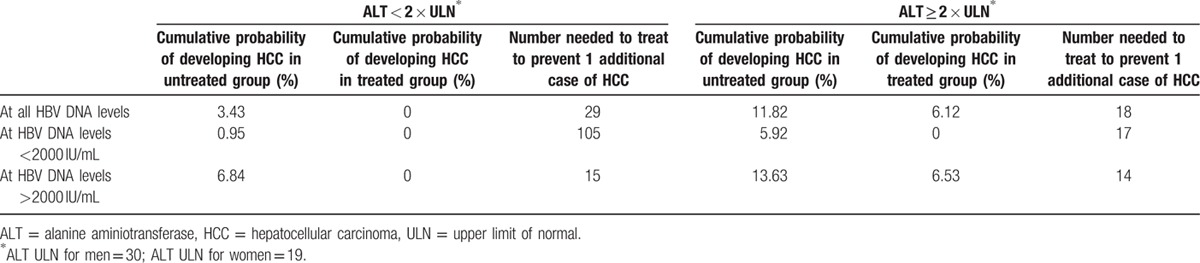
Number-needed-to-treat to prevent 1 incident case of hepatocellular carcinoma at 10 years of follow-up.

In patients with ALT ≥ 2 × ULN and HBV DNA > 2000 IU/mL (n = 403), crude HCC incidence was 1209.15 cases per 100,000 person-years in untreated patients versus 473.55 cases per 100,000 person-years in treated patients. Patients who were treated had a lower cumulative incidence of HCC compared to untreated patients (*P* = 0.0419). The cumulative probability of developing HCC was 3.83% in untreated group versus 1.89% in treated group, yielding a NNT of 52 patients at 5 years of follow-up (Table 5). At 10 years of follow-up, the cumulative probability of developing HCC was 13.63% in the untreated group, while the cumulative probability of developing HCC was 6.53% in the treated group, which yielded a NNT of 14 patients for this group (Table 6).

#### 3.3.2 ALT < 2 × ULN

Overall, crude HCC incidence was 314.46 cases per 100,000 person-years in untreated patients versus 0 cases per 100,000 person-years in treated patients. Patients who received treatment in this group had no incidence of HCC compared to some incident HCC in untreated patients (*P* = 0.0042). The cumulative probability of developing HCC was 1.23% in untreated group versus 0% in treated group, yielding a NNT of 81 patients at 5 years of follow-up (Table 5).

At 10 years of follow-up, the cumulative probability of developing HCC was 3.43% in the untreated group, while the cumulative probability of developing HCC was 0% in the treated group, which yielded a NNT of 29 patients (Table 6). The estimate of relative risk of HCC for antiviral treatment could not be estimated due to 0 HCC patients in the treated group.

In patients with ALT < 2 × ULN and HBV DNA > 2000 IU/mL (n = 1543), crude HCC incidence was 617.58 cases per 100,000 person-years in untreated patients versus 0 cases per 100,000 person-years in treated patients. For patients with ALT < 2 × ULN and HBV DNA < 2000 IU/mL, crude HCC incidence 85.02 cases per 100,000 person-years in untreated patients versus 0 cases per 100,000 person-years in treated patients. Patients who received treatment had a no cumulative incidence of HCC compared to untreated patients (*P* = 0.0013). The cumulative probability of developing HCC was 2.37% in untreated group versus 0% in treated group, yielding an NNT of 42 patients at year 5 follow-up (Table 5). At 10 years of follow-up, the cumulative probability of developing HCC was 6.84% in the untreated group, while the cumulative probability of developing HCC was 0% in the treated group, which yielded an NNT of 15 patients (Table 6). If both HBV DNA and ALT were low (<2000 IU/mL and <ULN, respectively), then the NNT was 105.

## 4 Discussion

In this cohort study of 3665 patients with CHB without cirrhosis, HCC incidence was significantly lower in patients treated with antiviral therapy in both ALT groups <2 × ULN or ≥2 × ULN following comprehensive adjustment for background risks with the previously validated REACH-B HCC risk score. Overall, antiviral therapy was independently associated with 72% reduction in HCC risk. Antiviral therapy has been shown to decrease the incidence of liver-related sequelae (cirrhosis, ESLD, and/or HCC) from CHB; however, the optimal time to initiate antiviral therapy has not been clearly defined, as evidenced by a diverse number of CHB treatment guidelines^[10–12,15,26]^ with current AASLD guideline recommending treatment only for those with elevated HBV DNA and ALT ≥ 2 × ULN.^[14]^ Two recently published reviews, 1 from Europe and 1 from the United States, the latter of which was a comprehensive systematic review, evaluated several studies of antiviral therapy in regards to HCC incidence, but there are currently no studies that specifically examine HCC risk in the CHB population without cirrhosis and with ALT < 2 × ULN.^[17,18]^

The natural history of CHB is quite dynamic, and ALT levels may fluctuate throughout the course of infection. Therefore, the presence of normal to minimally elevated ALT levels at 1 or few time points may not be an accurate representation of the extent of HBV-induced liver injury that may have accumulated overtime, leading to an underestimation of the degree of histologic damage and undertreatment of those who may benefit from therapy.^[16,27–31]^ To our knowledge, this is the first study to investigate the incidence of HCC and the effect of antiviral therapy in patients who may be at risk for HCC (age >40 years) but generally do not meet treatment criteria by the AASLD guideline: patients without cirrhosis and normal to mildly elevated ALT levels. In addition, we attempted to isolate the effect of treatment on the incidences of HCC in our comparison groups by using the REACH-B score to standardize all our cohorts onto the same risk scale.

In the present study, patients with ALT ≥ 2 × ULN who received treatment had a significantly lower incidence of HCC compared to those who were not treated, after adjustment for clinical and viral risk factors with REACH-B score (*P* = 0.0291). On subgroup analysis with patients with HBV DNA > 2000 IU/mL and ALT ≥ 2 × ULN, those who received treatment continued to have a significantly lower incidence of HCC compared to those who were not treated, after adjustment with REACH-B score (*P* = 0.0419). Our data are consistent with the current literature which suggest that patients with ALT ≥ 2 × ULN and HBV DNA > 2000 IU/mL would benefit from treatment compared to those who were not treated.^[9,14]^

In our study patients with ALT < 2 × ULN, inclusive of all levels of HBV DNA, we observed no incidence of HCC in patients who received treatment while some patients in the untreated group developed HCC. The current data suggest that patients with HBV DNA > 2000 IU/mL, 40 years or older may benefit from antiviral therapy regardless of ALT levels even in the absence of cirrhosis.

Although our findings highlight an important effect of treatment in patients with normal to minimally elevated ALT, our study is not without limitations, one of which is the lack of patient diversity, since the majority of our patients are Asians. As such, our results may not be generalizable to non-Asian patients. In addition, our study may not be applicable for patients under 40. A possible drawback to the REACH-B score is not factoring the adjustment for patients with cirrhosis. However, this limitation does not affect our study, as our cohort comprises only patients without cirrhosis. Because our study was retrospective in nature, we were not able to prospectively follow patients and collect regular serial ALT measurements, which may distinguish patients with persistently normal ALT versus those with fluctuating levels. However, according to results of a recent meta-analysis, even patients with only normal ALT or mildly elevated ALT may have significant histologic disease, generally defined as fibrosis stage 2 or higher.^[16,27,28]^ Recent data have also suggested that HBV DNA and quantitative HBsAg levels are more likely to be significant contributors to HCC risk than ALT.^[32]^ Results from the Study of E Antigen seRoCLearance of Hepatitis B and Elucidation of Risk Factors for Disease Control or Advancement in Taiwanese Hepatitis B carriers cohorts suggest that quantitative HBsAg and HBV DNA levels may be the more significant contributors to the risk of HCC and disease progression compared to ALT.^[32]^ Similarly, additional factors such as HBV genotype and presence of pre-S deletion or precore mutations can also play a role in HCC development but these data were not available for the vast majority of our cohort. Nevertheless, in our study, we were able to show that patients with HBV DNA levels above 2000 IU/mL, regardless of ALT levels (≥2 × ULN or <2 × ULN), had lower incidence of HCC when they were treated compared to patients who were not treated. In fact, the NNT to prevent 1 incident HCC at year 10 was only 14 to 15. Even in patients with HBV DNA < 2000 IU/mL, NNT was also fairly low at 17 if ALT was 2 × ULN or higher.

In summary, our findings of a treatment benefit in CHB patients without cirrhosis with ALT ≥ 2 × ULN are consistent with the literature, after appropriate adjustment for background risks using a previously validated HCC risk scoring system. However, we also observed significant HCC risk reduction with antiviral therapy in patients without cirrhosis who were older than 40 years with modestly elevated HBV DNA > 2000 IU/mL even if ALT < 2 × ULN, the population who currently may not meet criteria for treatment by some guidelines. The NNT for long-term HCC prevention was also low (only 14–15 by year 10) irrespective of ALT levels.
